# Rapid clinical detection of mycobacterial infections using M-Typer and flow-injection tandem mass spectrometry

**DOI:** 10.5588/ijtldopen.25.0323

**Published:** 2025-12-10

**Authors:** A. Głogowska, K. Majewski, B. Szulc, K. Kowalski, E. Augustynowicz-Kopeć

**Affiliations:** 1 Department of Microbiology, Institute of Tuberculosis and Lung Diseases, Warsaw, Poland;; 2 Centre of Clinical Medicine DiMedical Ltd., Lodz, Poland.

**Keywords:** tuberculosis, mycolic acids, *Mycobacterium*, mycobacterioses, FIA/MS-MS

## Abstract

**OBJECTIVES:**

We evaluated the utility of the M-Typer® assay, based on flow-injection tandem mass spectrometry mycolic acid profiling, in the diagnosis of TB and other mycobacterial infections.

**METHODS:**

The ability of the M-Typer® assay to identify *Mycobacterium* species was first evaluated using 40 clinical strains of known species. In the second phase, clinical specimens from 451 patients with suspected TB or non-tuberculous mycobacteria (NTM) infections were tested. Results were compared with those obtained from Xpert MTB/RIF and MGIT culture.

**RESULTS:**

The M-Typer® assay correctly classified all clinical strains as either *Mycobacterium tuberculosis* complex or NTM, and 80% were accurately identified to species level. In clinical specimens, it showed high sensitivity (94.4%) and specificity (93.63%) for TB diagnosis compared to Xpert MTB/RIF. Similar values were observed versus MGIT culture (sensitivity: 93.75%; specificity: 95.77%). However, the assay’s positive predictive value (PPV) was lower (70.8% vs. Xpert MTB/RIF; 45.45% vs. MGIT culture).

**DISCUSSION:**

The M-Typer® assay demonstrated diagnostic accuracy comparable to established methods, supporting its potential role in clinical microbiology for rapid, direct detection and species-level identification of mycobacteria. Nevertheless, its relatively low PPV indicates the need for further optimisation and validation in diverse clinical settings.

Mass spectrometry (MS) is an advanced analytical technique that enables to quantitatively analyse, identify, and characterise molecules on the basis of the mass-to-charge ratio. MS analyses are based on the generation of ions from the tested substance and their separation in mass analysers. This process allows to precisely determine the molecular weight of the tested substances and their quantity and structure.^1^ Due to its precision and versatile applicability, MS has been an essential research technique in scientific laboratories and various industries for many years.^2^ Standardisation of procedures and certification of equipment have facilitated its routine use in clinical diagnostics, including toxicology, therapeutic drug monitoring, endocrinology, and newborn screening.^3^ In recent years, MS has also revolutionised clinical microbiology by introducing matrix-assisted laser desorption/ionisation time-of-flight MS-based platforms (MALDI-TOF MS) to diagnostics.^4^ This approach enables rapid and precise identification of micro-organisms through proteomic analysis of constitutive protein profiles of bacteria, fungi, and mycobacteria isolated on culture media.^5^ In microbiology laboratories, MS is increasingly used not only for species-level identification but also for antimicrobial susceptibility testing, bacterial typing, toxin detection, and biomarker discovery in infectious diseases.^6^ Nowadays, a number of methods are used in routine microbiological diagnostics of TB and mycobacteria, and MS-based methods are only used to identify cultured mycobacteria. MALDI-TOF MS identification of mycobacteria relies on analysis of ribosomal proteins, whose extraction from the bacterial cell is not always easy. The thick cell wall of mycobacteria and the lower number of ribosomes and ribosomal proteins compared to other bacteria make the extraction of selected molecules for proteomic analysis time-consuming and require caution.^6–8^ This challenge has led many researchers to explore lipidomic approaches instead, focussing on species-specific lipid components detectable by MS.^8–11^ Mycobacteria have a unique lipid profile featuring mycolic acids (MAs), which are an attractive diagnostic marker. Diversity of MAs, including various chain lengths and chemical structures in particular *Mycobacterium* species, is widely used for their taxonomic classification.^9,10,12^

The use of liquid chromatography and then liquid chromatography coupled to MS (LC-MS) has recently contributed to tremendous success in the molecular characterisation of MAs. Today, LC-MS is a powerful tool for qualitative and quantitative determination of lipids, characterised with high specificity, sensitivity, repeatability, and high resolution capacity.^10^ Development of MS-based technology and determination of the mycobacterial lipidome are a sound base for development of diagnostic tests that allow to diagnose *Mycobacterium* rapidly and precisely. The CE-IVD M-Typer^®^ assay, developed by DiMedical, is one such innovative test. It detects and identifies MAs directly from clinical specimens from the respiratory system, as well as from other specimens such as urine, pleural fluid, and tissues. The test consists of three stages: extraction of mycobacterial MAs from clinical specimens, analysis of isolated MA by flow-injection tandem mass spectrometry (FIA-MS/MS) coupled to liquid chromatography, as well as analysis and profiling of the obtained spectra using dedicated M-Typer^®^ software. The procedure is simple, and results are available within 2 h.

This study aimed to evaluate usefulness of the M-Typer^®^ test in microbiological diagnostics of patients with TB and other mycobacterial infections.

## METHODS

In the first stage of the study, 47 bacterial cultures were analysed, including 40 clinical isolates from the *Mycobacteriaceae* family and 7 reference strains from other families of the order *Mycobacteriales*, in which MAs are one of the structural elements of the cell wall (Table 1). The strains were obtained from the collection of strains stored at the National Reference Laboratory for Mycobacteria in Warsaw, Poland. The species of the *Mycobacterium* spp. isolates were identified using GenoType assays (Hain Lifescience) before they were included. All strains were recovered from frozen aliquots and cultivated as liquid cultures under specific growth conditions. Positive cultures were subsequently analysed using the M-Typer® assay. The set of clinical isolates used in this study was entirely independent from the dataset used to train the M-Typer® classification algorithm.

**Table 1.** Bacterial strains analysed using the M-Typer® assay.

Species	Number of strains
*Mycobacterium tuberculosis*	1
*M. africanum*	1
*M. bovis*	2
*M. intracellulare*	2
*M. avium*	3
*M. chimaera*	5
*M. terrae*	1
*M. malmoense*	1
*M. kansasii*	2
*M. marinum*	2
*M. gordonae*	5
*M. flavescens*	1
*M. xenopi*	1
*M. fortuitum*	2
*M. peregrinum*	1
*M. mucogenicum*	1
*M. chelonae*	2
*M. abscessus* subsp. *abscessus*	2
*M. abscessus* subsp. *boletti*	2
*M. abscessus* subsp. *masiliense*	3
*Rhodococcus equi* PCM 559	1
*Nocardia asteroids* PCM 2138	1
*Corynebacterium glutamicum* PCM 1945	1
*Dietzia maris* PCM 2292	1
*Gordonia bronchialis* PCM 2167	1
*G. sputi* PCM 2144	1
*Tsukamurella paurometabola* PCM 2453	1
Total	47

PCM = Polish Collection of Microorganisms.

### Clinical specimens and study design

In the second stage of the study, a retrospective analysis of 451 clinical specimens collected between 2019 and 2022 from patients at the Regional Specialist Hospital of Tuberculosis, Lung Diseases and Rehabilitation in Lodz, Poland, was performed. The mean age of the patients was 57.2 years (±15.1), and 294 (65.2%) were males. Inclusion criteria comprised suspected TB or other mycobacterial infections, newly diagnosed cases, availability of mycobacteriological diagnostic data, and sufficient sample volume for additional M-Typer^®^ testing. Detailed clinical data such as clinical presentation of the disease, co-morbidities, symptoms, and radiological findings were not available at the time of analysis.

The analysed materials included specimens of sputum (349/77.4%), bronchial lavage (80/17.7%), pleural fluid (15/3.3%), broncho-aspirates (4/0.9%), urine (2/0.4%), and lung biopsy (1/0.2%). All materials were decontaminated with NaOH–N-acetyl-L-cysteine solution. After obtaining concentration by centrifugation, the sediment was re-suspended in 2 mL phosphate buffer (pH 6.8)^13^ and inoculated in a Mycobacterial Growth Indicator Tube (MGIT; Becton Dickinson [BD], Sparks, MD). Identification of isolated strains was determined by the TBc ID MGIT test (BD, Sparks, MD). Sediments were also subjected to microscopy with the Ziehl-Neelsen method using the BD BBL™ TB Stain Kit ZN (BD, Sparks, MD). Genetic testing was performed on 128 sediment samples using the Xpert MTB/RIF assay (Cepheid, Sunnyvale, CA). Testing was conducted in patients with suspected pulmonary TB based on clinical or radiological findings, in patients with positive smear microscopy, or in patients with a high suspicion of rifampicin resistance. The residual sediments were stored at −80°C until the M-Typer® test was conducted. An individual study number was assigned to each specimen, and patient identifiers were removed.

### Analysis by M-Typer® assay

Mycolic acids were isolated and analysed using FIA-MS/MS with the commercially available CE IVD M-Typer® Reagent kit (DiMedical, Lodz, Poland), following the manufacturer's instructions as detailed in the Supplementary Data. A preliminary data analysis was performed using Analyst® software (Sciex, Toronto, Canada), and detailed interpretation of MA profiles was conducted using dedicated M-Typer® software (DiMedical, Lodz, Poland). The M-Typer® algorithm outputs a ranked list of the top three most probable *Mycobacterium* species for each sample, accompanied by a percentage-based confidence score indicating the similarity of the sample's MA profile to reference profiles in the internal library. A full description of the methodology is provided in the Supplementary Data.

### Statistical analysis

Sensitivity, specificity, positive predictive value (PPV), negative predictive value (NPV), and 95% confidence intervals were calculated using PQStat software (PQStat Software, Poznan, Poland).

### Ethical statement

The study was approved by the Bioethics Committee of the Medical University of Lodz (Decision No.: RNN/785/13/KB). All samples were collected with informed consent and anonymised prior to analysis.

## RESULTS

The results of identifying 40 clinical *Mycobacterium* strains using the M-Typer® assay are summarised in Table 2. All isolates were correctly classified as either the *M. tuberculosis* complex (MTBC) or non-tuberculous mycobacteria (NTM) groups, with 4 strains (10%) identified as MTBC and 36 strains (90%) as NTM. Accurate species-level identification was achieved for 32 isolates (80%). In eight cases (20%), the M-Typer® assay did not identify the correct species as the top match; however, the correct species was still listed among the first three suggested matches, appearing in the second or third position (Table 2). A complete list of individual samples, including species identification, match ranking, and associated match probabilities, is provided in the Supplementary Data Table S2.

**Table 2.** Identification of *Mycobacterium* strains using the M-Typer® assay.

Species	Identification by the M-Typer® test		Number of strains	Notes
	MTBC/ NTM group	Species		
*M. tuberculosis*	MTBC	*M. tuberculosis*	1	
*M. africanum*	MTBC	*M. tuberculosis*	1	*M. africanum* in the 3rd match position
*M. bovis*	MTBC	*M. tuberculosis*	2	*M. bovis* in the 2nd match position
*M. intracellulare*	NTM	*M. intracellulare*	2	
*M. avium*	NTM	*M. avium*	3	
*M. chimaera*	NTM	*M. chimaera*	2	
*M. chimaera*	NTM	*M. intracellulare*	3	*M. chimaera* in the 2nd or 3rd match position
*M. terrae*	NTM	*M. terrae*	1	
*M. malmoense*	NTM	*M. malmoense*	1	
*M. kansasii*	NTM	*M. kansasii*	2	
*M. marinum*	NTM	*M. marinum*	1	
*M. marinum*	NTM	*M. avium*	1	*M. marinum* in the 2nd match position
*M. gordonae*	NTM	*M. gordonae*	5	
*M. flavescens*	NTM	*M. flavescens*	1	
*M. xenopi*	NTM	*M. xenopi*	1	
*M. fortuitum*	NTM	*M. fortuitum*	2	
*M. peregrinum*	NTM	*M. peregrinum*	1	
*M. mucogenicum*	NTM	*M. mucogenicum*	1	
*M. chelonae*	NTM	*M. chelonae*	1	
*M. chelonae*	NTM	*M. abscessus*	1	*M. chelonae* in the 2nd match position
*M. abscessus complex* ^A^	NTM	*M. abscessus*	7	
Total			40	

MTBC = *M. tuberculosis* complex; NTM = non-tuberculous mycobacteria.

A Including *M. abscessus* subsp. *abscessus* (two strains), *M. abscessus* subsp. *boletti* (two strains), and *M. abscessus* subsp. *masiliense* (three strains).

### Mycobacteriological diagnostic data from clinical samples

Among 451 clinical specimens, 1.8% (8) were Ziehl-Neelsen smear-positive and 3.5% (16) were culture-positive. All of them were identified as MTBC by the BD MGIT^™^ TBc Identification Test. Nine samples were excluded from the comparative analysis of the M-Typer® assay and culture results due to contamination with non-mycobacterial flora. The Xpert MTB/RIF test detected MTBC DNA in 3.9% (18) of specimens including 16 samples confirmed in MGIT culture. The M-Typer® assay was positive in 7.3% (33) of cases. In 24 cases, it detected MA profiles characteristic of *M. tuberculosis*, while in the remaining cases it was identified as *M. avium* (5), *M. chimaera* (2), *M. malmoense* (1), and *M. heamophilum* (1).

### Diagnostic performance of the M-Typer® assay

Comparative analysis of M-Typer® and Xpert MTB/RIF results was performed on 128 specimens and showed 94% consistency. The sensitivity of the M-Typer® test in detecting MTBC directly from clinical specimens appeared to be 94.4%, and the specificity was 93.63%. The PPV was 70.8%, whereas the NPV was 99% (Table 3). In 442 samples, M-Typer® and culture were consistent in 423 (96%) cases. Sensitivity and specificity of the M-Typer® test were 93.8% and 95.8%, respectively (PPV: 45.5%; NPV: 99.8%; see Table 4). According to the composite reference methods, 7 clinical specimens were classified as false-positives by M-Typer® when compared with Xpert MTB/RIF (Table 3) and 18 false-positives when compared with MGIT culture (Table 4).

**Table 3.** Diagnostic performance of the M-Typer® assay for detection of MTBC from clinical specimens in comparison with Xpert MTB/RIF test results.

		Xpert MTB/RIF		Sensitivity	Specificity	PPV	NPV
		Positive	Negative	95% CI	95% CI	95% CI	95% CI
M-Typer^®^	Positive^**A**^	17	7	94.4%	93.63%	70.8%	99.0%
	Negative	1	103	72.7–99.8	87.5–97.4	48.9–87.3	94.8–99.99

95% CI = 95% confidence interval; MTBC = *Mycobacterium tuberculosis* complex; PPV = positive predictive value; NPV = negative predictive value.

A All positive results detected by the M-Typer^®^ assay were identified as *M. tuberculosis*.

**Table 4.** Diagnostic performance of the M-Typer® assay for detection of mycobacterium in comparison with MGIT culture.

		MGIT culture		Sensitivity	Specificity	PPV	NPV
		Positive	Negative	95% CI	95% CI	95% CI	95% CI
M-Typer^®^	Positive	15	18	93.75%	95.77%	45.45%	99.75%
	Negative	1	408	69.7–99.8	93.4–97.4	28.1–63.6	98.6–99.9

95% CI = 95% confidence interval; PPV = positive predictive value; NPV = negative predictive value.

## DISCUSSION

Rapid and accurate diagnosis of mycobacterial infections is crucial to promptly initiate an effective treatment regimen, improve patient prognosis, and control further spread of the disease in the population.^14,15^ While the World Health Organization recommends molecular testing to detect MTBC directly from clinical samples, its high cost limits routine use.^16,17^ Affordable methods with comparable sensitivity and specificity are needed. MS-based methods offer a fast, precise alternative for identifying mycobacteria from cultures and clinical samples, including both TB and increasingly common atypical mycobacteria.^17,18^

This study evaluates the M-Typer® test, based on MA analysis via FIA-MS/MS, for detecting and identifying mycobacteria from cultures and clinical specimens. The M-Typer® assay demonstrated robust performance in differentiating between members of the MTBC and NTM group, with all tested strains accurately assigned to the correct group. At the species level, 80% of strains were correctly identified. However, in several cases, the difference in confidence scores between the two highest-ranked species was minimal, particularly among phylogenetically related organisms such as *M. avium* and *M. chimaera*, or *M. peregrinum* and other rapidly growing mycobacteria. This outcome reflects the high biochemical and lipidomic similarity among certain species within the *Mycobacterium* genus. Species belonging to the *M. avium* complex (MAC), as well as those within the MTBC, exhibit significant genomic and phenotypic similarities. Such close relatedness poses a challenge for classification models based solely on lipidomic data. An example confirming this fact is the case of seven strains belonging to the MTBC and MAC for which the M-Typer® assay did not unequivocally identify the correct species; however, the correct species was still listed among the top three suggestions, typically in the second or third match position. These findings underline the importance of contextual interpretation in cases with low inter-species discrimination and support the need for further enrichment of the reference library with additional isolates representing these species groups.

Comparative studies on lipid profiles of *M. marinum* and MAC species are limited, but observed cross-reactivity suggests similarities needing further investigation. The current algorithm used in the M-Typer® assay cannot distinguish between *M. abscessus* subspecies due to their overlapping MA profiles and the limited number of isolates in the algorithm’s library. While this limitation does not affect genus- or species-level classification, accurate identification at the subspecies level is critical in clinical practice.^19^ Future development and continuous enrichment of the spectral database are expected to improve discriminatory performance at the subspecies level.

Our analysis conducted on clinical specimens showed that the M-Typer® test exhibits high sensitivity and specificity compared to the Xpert MTB/RIF test (94.4% and 93.63%) and MGIT culture (93.75% and 95.77%). Although M-Typer® is a new test on the diagnostic market and our study is the first analysis of results using this test, several reports describing similar research approaches can be found in literature.^12,20^ Shui et al.^20^ reported using electrospray ionisation MS to detect mycobacterial MAs directly in clinical samples, achieving 94% sensitivity and 93% specificity for TB diagnosis. Szewczyk et al.^12^ noted much lower sensitivity (69%) for detecting mycobacterial MAs directly from sputum using LC-MS/MS, which increased to 94% after 10 days incubation in Middlebrook 7H9 medium. This approach detected MAs in patients with radiologically confirmed TB but negative standard bacteriology, though one false-negative occurred.

In our study, discrepancies in the two methods were observed in 19 specimens (4.2%), 17 of which referred to both genetic and culture methods. One false-negative M-Typer® result was found, matching both comparison methods. The sample was Ziehl-Neelsen-negative, and the patient was likely paucibacillary, which may have contributed to the lower MA concentration and subsequent false-negative result. In seven cases, the M-Typer® assay allowed to detect MAs characteristic of mycobacteria, while the Xpert MTB/RIF test and culture method failed to confirm the presence of *M. tuberculosis* in the tested specimens. A similar observation was made for nine specimens from which no culture was obtained, and the M-Typer® test detected and identified MAs characteristic of NTM. Due to the lack of availability of detailed clinical and radiological data on the study population, the above results should be interpreted as false-positives and highlight the need for further prospective studies. However, the observed false-positive results may be attributed to differences in the detection thresholds of the diagnostic methods employed. The M-Typer® test demonstrated a significantly lower detection threshold, approximately 20 times lower than the Xpert MTB/RIF assay (<6 vs. 130 CFU/mL) and more than twice as low as the Xpert MTB/RIF Ultra assay (16 CFU/mL).^21,22^

One of the key limitations of our study is the relatively small number of true-positive TB cases confirmed by reference methods (17 samples), which resulted in wide confidence intervals and limits the statistical power of the presented sensitivity estimates. The use of the Xpert MTB/RIF Ultra in this study might have increased the number of true-positive cases detected.

## CONCLUSION

This study clearly indicates that the M-Typer® test can be a valuable complement to current diagnostic methods. The test is characterised by high sensitivity and specificity for the diagnosis of pulmonary TB, comparable to those of established diagnostic methods such as Xpert MTB/RIF and culture. Therefore, its future integration into the current TB diagnostic regimen and using it as a supportive tool for taking clinical decisions are worth considering. Application of the M-Typer® test to detect drug-resistant mycobacteria in the future would be a huge advance in diagnosis of patients with TB. Currently, a rapid test for the direct detection of NTM is an urgent need, and the M-Typer® assay has a great potential but requires further evaluation in confirmed mycobacteriosis cases, as none were identified in the present study. Additionally, some NTM species cause extra-pulmonary symptoms, so future studies should include a broader range of clinical samples. In our study, all 17 extra-pulmonary specimens (3.7%) tested negative. The M-Typer® test is quick and easy to perform and requires minimal amounts of clinical specimens and reagents, which makes it inexpensive. The M-Typer® assay requires BSL-3 conditions for initial sample processing, including decontamination and lipid extraction, due to the risk of infectious mycobacteria. After chemical inactivation, mass spectrometric analysis can be safely performed under BSL-2 conditions. A high price of a mass spectrometer and, in subsequent years, high maintenance costs might be the only limitations to implementing the test into routine diagnostics.

## Supplementary Material


