# Supplementary material for: Rapid clinical detection of mycobacterial infections using M-Typer and flow-injection tandem mass spectrometry
Source: IJTLD Open. 2025 Dec 10;2(12):751–6. doi: 10.5588/ijtldopen.25.0323 (PMC12699965; doi:10.5588/ijtldopen.25.0323)
Supplement: Supplementary file 1 [file ijtldopen25-0323_supplementarydata1.pdf]

Supplementary materials

1. Methods:

Mycolic acid extraction

Mycolic acid extract sample preparation was performed using M-Typer® Reagent kit (DiMedical, Lodz, Poland) and consisted of 3 main steps: alkaline hydrolysis at elevated temperature, neutralization and liquid-liquid extraction.Clinical specimens were subjected to a standardized decontamination procedure using N-acetyl-L-cysteine and NaOH, as described in the main manuscript. After centrifugation, the resulting pellet was washed with the dedicated wash reagent included in the M-Typer® Reagent Kit prior to mycolic acid extraction. Cultured strains, grown in liquid media, were processed without the decontamination step but were also subjected to the washing step before extraction. Alkaline hydrolysis was carried out in a water bath at 90°C for 60 minutes. After the solution cooled, neutralization was carried out using a concentrated acid solution at a volume ratio of 1:1 with precipitation of an insoluble salt. The resulting solution was then subjected to liquid-liquid extraction at a volume ratio of 2:1 conducted for 30 minutes on a rotator-type shaker. The lower organic phase was then collected into a chromatography vial and secured with a Teflon-coated septum cap and then assigned for storage at -80°C.

FIA-MS/MS analysis

Analysis of mycolic acid profiles by flow injection tandem mass spectrometry (FIA-MS/MS) was performed on a QTRAP® 6500+ tandem mass spectrometer (Sciex, Torronto, Canada) coupled to an ExionLC AD liquid chromatograph (Sciex, Torronto, Canada). In the course of the study, full compatibility (interchangeability) of M-Typer® technology (DiMedical, Lodz, Poland) was proven on a Triple Quad LCMS-8060 system coupled to an LC-20AD chromatograph (Shimadzu, Kyoto, Japan). The analysis was carried out in negative

electrospray ionization (ESI), and the unique signals of mycolic acids were detected and monitored using single reaction monitoring (SRM) mode. The test includes monitoring of 52 SRM transitions, where each transition corresponds to a different mycolic acid (MAs). The SRM transitions are selected to account for both the molecular weight of the MAs (Q1 mass) and the length of its a-alkyl chain (Q3 mass). The collection of 52 SRM transitions takes into account a wide range of mycolic acid masses from 887 to 1295 Da, differences in the alpha-alkyl chain lengths of C22, C24 and C26, and considering all classes of MAs: alpha, methoxy and mycolic keto acids.

FIA-MS/MS analysis was carried out using reagents (mobile phases and autosampler wash reagent) included in the M-Typer Reagent Kit (DiMedical, Lodz, Poland). A sample of mycolic acid extract (50ul) was injected directly into the flow on the ESI-MS/MS system. The gradient of the flow and mobile phase composition was chosen to provide the desired "hump-like" shape of the SRM signals while preventing carryover effects between sequential analyses. The time for a single analysis was 1min/sample and the time between consecutive injections was 1min/sample.

Mycolic acids profile analysis using M-Typer® library

The obtained measurement data is retrieved, normalized and then the similarity of the obtained MAs profiles is compared with the MAs profiles of reference strains and characterized clinical isolates contained in the library. During the readout, the absolute average intensity within the hump-shaped signal for each SRM transition is evaluated. The data are then normalized by determining the percentage ratio of the intensity of a given SRM transition relative to the most intense SRM transition in the profile under study. The normalized profiles collectively form the MAs profile. The MAs profile is then analyzed by comparing the studied profile against all profiles contained in the library. The library contains more than 1300 MAs profiles collected on different strains, species, subspecies and mycolic

acid concentration (bacterial suspension density). Detailed information on the composition of the mycobacteria included in the library can be found in the table below (Table 1s). The similarity of the obtained MAs profiles to those in the library is expressed in percentages and presented as the strain with the highest matching value.

Table 1s. Reference *Mycobacterium* strains/isolates included in the training set for algorithm development.

Group	Species	Reference strains	Clinical isolates (n)
Mycobacterium tuberculosis complex (MTBC)	*M. africanum*	*M. africanum* NC 14049	0
	M. bovis	*M. bovis* NC10772<br>*M. bovis subsp. caprae* NC 13882	2
	M. bovis BCG	-	2
	M. canettii	*M. canettii* NC 13887	-
	M. microti	*M. microti* NC 08710	-
	M. pinnipedii	-	1
	M. tuberculosis	*M. tuberculosis* NC 13144<br>*M. tuberculosis* H37Rv ATCC 25618	39
Mycobacterium avium complex (MAC)	*M. avium*	*M. avium subsp. avium* ATCC 25291<br>*M. avium subsp. silvaticum* ATCC 49884<br>*M. avium subsp. paratuberculosis* ATCC 43544	29
	M. chimerae	*M. chimaera* NCTC 13934<br>*M. chimaera* NCTC 13933<br>*M. chimaera* NCTC 13781	6
	M. intracellurae	*M. intracellulare* ATCC 13950	28
Non-tuberculosis mycobacteria (NTM)	*M. abscessus*	*M. abscessus* ATCC 23045<br>*M. abscessus subsp. massiliense* DSM45103	11
	M. celatum	-	1
	M. chelonae	*M. chelonae* ATCC 35752	7
	M. flavescens	-	1
	M. fortuitum	*M. fortuitum subsp. fortuitum* ATCC 6841<br>*M. fortuitumsubsp. margeritense* ATCC BAA-2397<br>*M. fortuitum subsp. acetamidolyticum* ATCC 35931<br>*M. fortuitum subsp. thermophilum* ATCC 27408	20
	M. gordonae	*M. gordonae* ATCC 14470	25
	M. haemophilum	*M. haemophilum* ATCC 29548	-
	M. interjectum	-	1

	M. kansasii	*M. kansasii* ATCC 12478	28
	M. lentiflavum	-	4
	M. malmoense	*M. malmoense* ATCC 29571	3
	M. marinum	-	1
	M. mucogenicum	-	1
	M. peregrinum	-	1
	M. szulgai	*M. szulgai* ATCC 35799	-
	M. scrofulaceum	*M. scrofulaceum* ATCC 19981	-
	M. shimoidei	*M. shimoidei* NC 13909	0
	M. simiae	*M. simiae* ATCC 25275	1
	M. smegmatis	*M. smegmatis* ATCC 19420	-
	M. terrae	*M. terrae* ATCC 15755	-
	M. xenopii	*M. xenopi* ATCC 19250	13

Table 2s. Identification of *Mycobacterium* strains using the M-Typer® assay, including the degree of match.

Species	M-Typer® identification result	
	**Species**	**%**
M. tuberculosis	***Mycobacterium tuberculosis***	94,88
	Mycobacterium africanum	85,47
	Mycobacterium bovis	74,04
M. africanum	***Mycobacterium tuberculosis***	90,75
	Mycobacterium bovis	81,5
	Mycobacterium africanum	68,12
M. bovis	***Mycobacterium tuberculosis***	90,75
	Mycobacterium bovis	74,7
	Mycobacterium africanum	68,51
M. bovis	***Mycobacterium tuberculosis***	90,83
	Mycobacterium bovis	73,41
	Mycobacterium africanum	68,87
M. intracellulare	***Mycobacterium intracellulare***	90,3
	Mycobacterium chimaera	76,22
	Mycobacterium avium	69,73
M. intracellulare	***Mycobacterium intracellulare***	93,96
	Mycobacterium chimaera	87,25
	Mycobacterium avium	83,93
M. avium	***Mycobacterium avium***	88,27
	Mycobacterium intracellulare	88,19
	Mycobacterium chimaera	85,71
M. avium	***Mycobacterium avium***	88,48
	Mycobacterium intracellulare	85,09
	Mycobacterium chimaera	81,62
M. avium	***Mycobacterium avium***	91,76
	Mycobacterium intracellulare	88,39
	Mycobacterium chimaera	83,94
M. chimarea	***Mycobacterium chimaera***	89,94
	Mycobacterium intracellulare	89,13
	Mycobacterium avium	76,92
M. chimarea	***Mycobacterium chimaera***	89,94
	Mycobacterium intracellulare	89,13
	Mycobacterium avium	76,92
M. chimarea	***Mycobacterium intracellulare***	91,3
	Mycobacterium chimaera	87,58
	Mycobacterium avium	81,13
M. chimarea	***Mycobacterium intracellulare***	93,27

	Mycobacterium avium	89,79
	Mycobacterium chimaera	89,12
M. chimarea	***Mycobacterium intracellulare***	85,49
	Mycobacterium chimaera	85,25
	Mycobacterium avium	81,22
M. terrae	***Mycobacterium terrae***	63,35
	Mycobacterium mucogenicum	37
	Mycobacterium intracellulare	36,98
M. malmoense	***Mycobacterium malmoense***	86,38
	Mycobacterium celatum	85,84
	Mycobacterium simiae	42,54
M. kansasii	***Mycobacterium kansasi***	96,71
	Mycobacterium gordonae	86,64
	Mycobacterium intracellulare	85,66
M. kansasii	***Mycobacterium kansasi***	93,38
	Mycobacterium intracellulare	86,82
	Mycobacterium gordonae	80,51
M. marinuum	***Mycobacterium marinum***	52,26
	Mycobacterium kansasi	37,59
	Mycobacterium gordonae	31,91
M. marinum	***Mycobacterium avium***	44,51
	Mycobacterium marinum	41,82
	Mycobacterium kansasi	40,81
M. gordonae	***Mycobacterium gordonae***	89,7
	Mycobacterium kansasi	56,31
	Mycobacterium avium	41,23
M. gordonae	***Mycobacterium gordonae***	93,51
	Mycobacterium kansasi	62,18
	Mycobacterium schulgai	48,99
M. gordonae	***Mycobacterium gordonae***	86,73
	Mycobacterium kansasi	79,74
	Mycobacterium intracellulare	52,49
M. gordonae	***Mycobacterium gordonae***	95,3
	Mycobacterium kansasi	70,33
	Mycobacterium schulgai	53,35
M. gordonae	***Mycobacterium gordonae***	85
	Mycobacterium kansasi	69,58
	Mycobacterium schulgai	49,94
M. flavescens	***Mycobacterium flavescens***	54,87
	Mycobacterium intracellulare	52,95
	Mycobacterium mucogenicum	49,25

M. xenopi	***Mycobacterium xenopi***	97
M. fortuitum complex	***Mycobacterium fortuitum***	76,22
	Mycobacterium peregrinum	66,92
	Mycobacterium intracellulare	39,68
M. fortuitum	***Mycobacterium fortuitum***	92,7
	Mycobacterium fortuitum margeritense	91,14
	Mycobacterium avium	70,37
M. peregrinum	***Mycobacterium peregrinum***	86,61
	Mycobacterium fortuitum	86,48
	Mycobacterium avium	50,36
M. mucogenicum	***Mycobacterium mucogenicum***	42,21
	Mycobacterium smegmatis	22,39
	Mycobacterium flavescens	20,03
M. chelonae	***Mycobacterium chelonae***	92,68
	Mycobacterium abscessus	77,7
	Mycobacterium avium	52,86
M. chelonae	***Mycobacterium abscessus***	90,84
	Mycobacterium chelonae	87,16
	Mycobacterium avium	60,42
M. abscessus complex (M. abscessus subsp. abscessus)	***Mycobacterium abscessus***	93
	Mycobacterium chelonae	80,58
	Mycobacterium avium	48,14
M. abscessus complex (M. abscessus subsp. abscessus)	***Mycobacterium abscessus***	92,93
	Mycobacterium chelonae	80,35
	Mycobacterium avium	45,54
M. abscessus complex (M. abscessus subsp. boletti)	***Mycobacterium abscessus***	95,49
	Mycobacterium chelonae	87,1
	Mycobacterium avium	53,93
M. abscessus complex (M. abscessus subsp. boletti)	***Mycobacterium abscessus***	94,23
	Mycobacterium chelonae	86,61
	Mycobacterium avium	51,89
M. abscessus complex (M. abscessus subsp. masiliense)	***Mycobacterium abscessus***	96,14
	Mycobacterium chelonae	82,36
	Mycobacterium avium	58,9
M. abscessus complex (M. abscessus subsp. masiliense)	***Mycobacterium abscessus***	88,24
	Mycobacterium chelonae	82,32
	Mycobacterium avium	57,78
M. abscessus complex (M. abscessus subsp. masiliense)	***Mycobacterium abscessus***	94,61
	Mycobacterium chelonae	86,23
	Mycobacterium avium	56,92
